# Aquaporin-4: A Potential Therapeutic Target for Cerebral Edema

**DOI:** 10.3390/ijms17101413

**Published:** 2016-09-29

**Authors:** Guanghui Tang, Guo-Yuan Yang

**Affiliations:** Neuroscience and Neuroengineering Research Center, Med-X Research Institute and School of Biomedical Engineering, Shanghai Jiao Tong University, Shanghai 200030, China; shtangguanghui@163.com

**Keywords:** aquaporin-4, brain edema, therapeutic target, water channel

## Abstract

Aquaporin-4 (AQP4) is a family member of water-channel proteins and is dominantly expressed in the foot process of glial cells surrounding capillaries. The predominant expression at the boundaries between cerebral parenchyma and major fluid compartments suggests the function of aquaporin-4 in water transfer into and out of the brain parenchyma. Accumulating evidences have suggested that the dysregulation of aquaporin-4 relates to the brain edema resulting from a variety of neuro-disorders, such as ischemic or hemorrhagic stroke, trauma, etc. During edema formation in the brain, aquaporin-4 has been shown to contribute to the astrocytic swelling, while in the resolution phase, it has been seen to facilitate the reabsorption of extracellular fluid. In addition, aquaporin-4-deficient mice are protected from cytotoxic edema produced by water intoxication and brain ischemia. However, aquaporin-4 deletion exacerbates vasogenic edema in the brain of different pathological disorders. Recently, our published data showed that the upregulation of aquaporin-4 in astrocytes probably contributes to the transition from cytotoxic edema to vasogenic edema. In this review, apart from the traditional knowledge, we also introduce our latest findings about the effects of mesenchymal stem cells (MSCs) and microRNA-29b on aquaporin-4, which could provide powerful intervention tools targeting aquaporin-4.

## 1. Introduction

Cerebral edema is a severe clinical complication and characterized by the pathological swelling of brain tissue due to the increase of brain water content. It progresses in many types of brain insults, such as trauma [1], ischemia [2], tumors [3] and inflammation [4]. The consequences of cerebral edema are lethal due to the elevated intracranial pressure and the compromised cerebral blood flow (CBF). Water disturbances are known to be critical in the progression of cerebral edema [5]. Despite the clinical significance of edema, little is known about the mechanisms that are responsible for the brain water transport and homeostasis. As a result, current therapeutic strategies are limited to decompressive craniectomy and intravascular administration of hyperosmolar agents that were introduced more than 70 years ago.

A family of water channel proteins called aquaporins, which has been identified in mammals, may offer a mechanism explanation for brain edema and thus provide therapeutic options. In 1988, aquaporin-1 was purified and identified in human erythrocytes as the first family member of aquaporins [6]. Up to now, more than 10 aquaporins have been discovered in mammals [7]. In the central nervous system, aquaporin-4 is the most important type of aquaporin and mediates the water homeostasis in normal or pathophysiological conditions. The aim of this review is to look into: (1) the role of aquaporin-4 in the development of cerebral edema; (2) the potential of aquaporin-4 as a therapeutic target in edema with our new findings.

## 2. Brain Edema

Brain edema, as a consequence of various brain insults, including traumatic brain injury, stroke and brain tumor, exacerbates the development of these diseases and is a major cause of morbidity and death in these patients [8]. The pathophysiological swelling of brain tissue is a main feature of cerebral edema due to the progressive increase in water content [9]. A logical explanation to understanding the mechanisms of cerebral edema formation was first carried out by Igor Klatzo, where he classified edema into cytotoxic and vasogenic types [10]; a third type, named hydrocephalic edema or interstitial edema, has subsequently been described [11].

### 2.1. Cytotoxic Edema

Cytotoxic edema is characterized by the intact blood-brain barrier (BBB), but a dysfunction of the sodium and potassium pump in the cell membrane, leading to a shift of water from the interstitial into the intracellular compartment and a net uptake of water from blood into brain parenchyma [12,13].

In several pathological cerebral edemas, deprivation of nutrients and oxygen leads to the sodium-potassium pump failure on astrocytic cell membranes [12,14]. As a consequence, a massive accumulation of sodium ions occurs intracellularly. Because of the osmotic change, cells uptake net water and subsequently swell [15]. It is these swollen individual cells in the brain that act as the main characteristic to distinguish cytotoxic edema and vasogenic edema. Among all of the swollen cell types, astrocyte swelling may be the most important early signal predisposing the brain to further damage [16].

In addition to the dysfunction of sodium-potassium pumps, aquaporin-4 dysregulation is also involved in mediating cytotoxic edema in astrocytes [17,18]. For instance, after focal brain ischemia, aquaporin-4 is observed to lose polarized localization to astrocytic end-feet, which is associated with the loss of the astrocyte end-foot anchorage protein β-dystroglycan (DG). Furthermore, agrin-deficient mice lack polarized localization of both β-DG and aquaporin-4 at astrocytic end-feet and do not develop cytotoxic edema after ischemia [19].

### 2.2. Vasogenic Edema

BBB integrity disruption contributes significantly to the development of vasogenic edema, which directly results from tight junction breakdown between vascular endothelial cells [20]. As a result, fluid and proteins from the vasculature penetrate into the interstitial space and cause expansion of the brain extracellular compartment [21]. If there is no interference, it leads to elevated intracranial pressure, reduced cerebral blood flow and, ultimately, cerebral herniation and death [5].

It seems that gray matter is more resistant to the influx than white matter where fluid accumulation takes place first, which explains why vasogenic edema fluid is just found in the white matter when scanned by CT or MRI [22]. Moreover, this type of edema has usually been seen in the late stages of cerebral ischemia, further inducing focal cerebral blood flow (CBF) reduction, cell necrosis and apoptosis [12]. Mechanisms contributing to vasogenic edema include physical crash by arterial hypertension or trauma and tumor-released vasoactive or endothelial destructive compounds (e.g., arachidonic acid, excitatory neurotransmitters, eicosanoids, bradykinin, histamine and free radicals) [3,23].

In most situations, there is no clear border to discriminate cytotoxic and vasogenic edema. For instance, even though it is well accepted that cytotoxic edema is the prevalent event right after ischemic stroke, vasogenic edema is also concomitant. The use of MRI and CT techniques allows for the discrimination and shows that in the case of ischemic stroke, the cytotoxic type is dominant in the acute phase [22].

### 2.3. Hydrocephalic Edema or Interstitial Edema

Damaged absorption of cerebrospinal fluid (CSF) increases trans-ependymal CSF flow, which subsequently leads to acute hydrocephalus. Although plenty of pathological events are associated with CSF flow obstruction, the dilatation of the cerebral ventricles is the most common consequence [22].

Clinically, the surgical intervention is by the shunting of CSF from the lateral ventricle into another part of the body where it can be absorbed. This strategy involves the implantation of two silicone rubber tubes and a valve system to drain the excess CSF from the ventricles so that the elevated intracranial pressure can be relieved [24].

## 3. Aquaporin-4 Structure and Distribution

Aquaporins are a family of channel proteins that facilitate water transport. They are well documented to express on the cytosolic membranes and are conserved from bacteria to animal cells [25]. Structural analysis showed that a pore is present in the center of each aquaporin molecule [26]. Furthermore, Asn-Pro-Ala (NPA) motifs, as well as the electrostatic field among aquaporins are highly conserved, suggesting a role to prevent proton conduction [27]. Among all of the aquaporins, aquaporin-4, expressing on the end-feet of astrocytes, has been under extensive investigation and is emerging as a novel therapeutic target in cerebral edema intervention.

Aquaporin-4 is composed by homo-tetramers with each monomer representing a water channel. Meanwhile, a pore is formed in the center of the four monomers, which probably promotes the flow of water, cations and gases [28].

Aquaporin-4 is abundant in the brain and spinal cord, where its distribution pattern is highly polarized, with intensive expression in astrocyte end-feet that surround capillaries [29]. Nonetheless, in pathological conditions like brain tumors, trauma or inflammation, it is redistributed to membrane domains apart from end-feet areas. This dislocation is due to the degradation of the proteoglycan agrin by the matrix metalloproteinase 3 [18,19]. Although aquaporin-4 is ubiquitously expressed in kidney, airways, stomach and colon, global genetic deletion mice do not show abnormality in growth or tissue morphology, which could be explained by the compensatory expression of its counterpart aquaporins [30].

Despite the well-studied role in water movement, aquaporin-4 may also benefit cell adhesion. Through electron crystallography analysis, a short extracellular helix is discovered in aquaporin-4, which interacts weakly, but specifically with aquaporin-4 molecules in adjacent membranes. To confirm this putative idea, aquaporin-4 was overexpressed in cells, and were found to be clustered [27]. In addition, aquaporin-4 probably participates in glial scar formation after brain injury by promoting glial cells’ migration [31]. Collectively, these data underline the versatile and complex functions of aquaporin-4.

## 4. Aquaporin-4 in Brain Edema

### 4.1. Versatile Function of Aquaporin-4

Aquaporin-4 is found to be overexpressed in the reactive astrocytes after cerebral injury [32]. As an important water channel protein, its upregulation or redistribution is associated with edema formation in the acute phase. In a focal ischemic stroke model, aquaporin-4-deficient mice have shown improved neurological outcome, decreased cerebral edema and hemispheric enlargement at 24 h [33]. However, in other studies, aquaporin-4 deletion led to greater edema load [34]. The key to understating the apparent discrepancy is the roles of bidirectional water transport, which enable it to contribute to both edema formation at early time points and the clearance of water from the brain into blood vessels at later time points [17].

In injuries that create cytotoxic edema in the absence of BBB breakdown, aquaporin-4 is a major contributor. Its upregulation and redistribution in trauma, pneumococcal meningitis, glioblastoma or ischemia were associated with worse outcome [16,18,19,35,36], while edema was reduced with aquaporin-4 downregulation or deletion [33]. Through overexpression in glial cells, it was directly revealed that aquaporin-4 is a rate-limiting step in the formation of ionic edema [37].

A possibility contributing to the brain edema alleviation in aquaporin-4-deficient mice is the reduced apoptotic astrocytes, which is a consequence of the reduced swollen astrocyte. We demonstrated that downregulation of aquaporin-4 could efficiently reduce astrocyte apoptosis after ischemic injury. The in vitro data showed the same working model [16].

In vasogenic edema conditions where blood extravasations in parenchyma need to be cleared by bulk flow into CSF compartments and veins [38], aquaporin-4 seems to be a crucial player as the deficient animals displayed an impaired ability to efficiently clean the edema [39].

Aquaporin-4 not only serves as a water influx route, but also initiates downstream astrocytic Ca^2+^ signaling events. Through activation of P2 purinergic receptors in astrocytes, brain swelling triggers Ca^2+^ signaling that may potentially exacerbate brain edema. In addition, osmotic stress was observed to mediate ATP release from cultured astrocytic cells in an aquaporin-4-dependentmanner [40], which may affect the pathological outcomes, as well.

In cultured astroglial cells, aquaporin-4 was polarized to the membrane leading edge, a process related to rapid formation and retraction of plasma membrane protrusions, such as lamellipodia and filopodia [41,42], implying an involvement of aquaporin-4 in the migrating cells. As a critical event in the later phase after brain injury, the glial scar formation also involves the upregulation of aquaporin-4 [31]. Increased aquaporin-4 expression, together with its polarization to the leading edge may further contribute to the glial scar formation.

Even though aquaporin-4 is well known to be expressed in astrocytes in brain, Tomas-Camardiel with his colleagues demonstrated that activated microglia strongly expressed aquaporin-4 in response to LPS stimulation [43], which represents a molecular adaptation in maintaining water and ion homeostasis and may also contribute to the proliferation and migration of microglial cells in injured brain. Collectively, these data suggest the complexity of aquaporin-4 in pathological settings.

### 4.2. Aquaporin-4 and Neuroinflammation

Neuroinflammation is a concomitant syndrome that has been demonstrated to significantly contribute to the progression of various brain injuries, including traumatic brain injury and ischemic or hemorrhagic stroke, among others [12,20,44]. Aquaporin-4 expression was found upregulated during the edema build-up and resolution phases in focal brain inflammation [16,45]. In an inflammatory model that was created by the injection of l-a-lysophosphatidylcholine (LPC) stearoyl, a slight upregulation of aquaporin-4 was observed during the build-up phase, but followed by a robust transcriptional and translational level of aquaporin-4 expression during the edema resolution phase [45]. In another inflammatory model induced by LPS, strong expression of aquaporin-4 mRNA and protein in response to LPS injections was observed, as well [43]. 

We have also found the association of inflammatory cytokines’ (IL-1β, IL6 and TNF-α) production and aquaporin-4 expression in acute ischemic stroke, which implies an intrinsic relationship between them. We further explored the relationship between inflammatory cytokines and aquaporin-4 and clarified that it is the inflammatory cytokines released from the microglia that are responsible for the induction of aquaporin-4 [16].

Taken together, these data suggest that aquaporin-4 is an inflammatory mediator [46] and plays a differential role during edema build-up and resolution. In the build-up phase, aquaporin-4 overexpression may result in the swelling and death of astrocytes and activation of microglia, which could be the main contributor to BBB disruption that leads to plasma protein leakage and extravascular fluid accumulation. On the contrary, during the resolution phase, it perhaps drives water extravasations from the brain parenchyma to CSF and blood vessels. Thus, we could cautiously state that aquaporin-4 plays a negative role during edema formation, while it takes a positive part in the resolution phase (Table 1).

## 5. Aquaporin-4 as a Therapeutic Target in Cerebral Edema

An important goal in brain injury treatment is the control and reduction of cerebral edema. As aquaporin-4 has shown a critical role in the progression of cerebral edema, it could be a promising target for the intervention.

### 5.1. Aquaporin-4 Inhibitors

Several pharmaceutical chemicals targeting at aquaporin-4 have shown positive effects on cerebral edema in both humans and animals. Vincent J. Huber and his colleagues identified arylsulfonamides as aquaporin-4 inhibitors, among which acetazolamide (AZA) was found to be the most efficient one with an 80% water transport inhibition [52]. They subsequently proved that some antiepileptic drugs (AEDs), such as topiramate (TPM) and zonisamide (ZNS), which share a variety of physiochemical properties with AZA, were efficient in aquaporin-4 inhibition, as well, which may partly contribute to their antiepileptic function [53]. This group further identified a novel aquaporin-4 inhibitor called 2-(nicotinamide)-1,3,4-thiadiazole (TGN-020). In a brain ischemic model, they found significant reductions in the cerebral edema and cortical infarction size in the pretreated mice [54].

Erythropoietin (EPO), the major hemopoietic growth factor, has been widely used as an efficient neuro-protective agent [55,56,57,58]. The beneficial effects of EPO are partially due to the antagonist of the glutamate-mediated water flux. Using a multifaceted model of water permeability in aquaporin-4-positive astrocytes, the mGluR-mediated water permeability increase was abolished in the EPO administration condition prior to water intoxication [59]. These findings indicate that EPO interacts with aquaporin-4 and reduces the risk of astrocyte swelling in brain insults, showing a positive role in the treatment of cerebral damage associated with water disturbances.

Since inflammation has been shown to be an underlying aquaporin-4 inducer, any drugs that repress the inflammation response could be potential aquaporin-4 inhibitors. CXCL12 is well known as a key chemokine for leukocyte recruitment by interacting with its receptor CXCR4 during brain ischemia [20,60]. A CXCR4 antagonist drug called AMD3100 significantly suppressed inflammatory response and reduced BBB disruption in the context of ischemic stroke [20]. Besides, a renowned traditional Chinese medicine Tongxinluo was found to benefit BBB integrity by reducing the inflammatory cytokines’ expression, as well, in mice subjected to middle cerebral artery occlusion [12]. The beneficial effects on brain edema may at least partially be attributed to the block of the aquaporin-4 upregulation through the attenuated cytokines’ release.

### 5.2. Other Strategies

**Figure 1:**
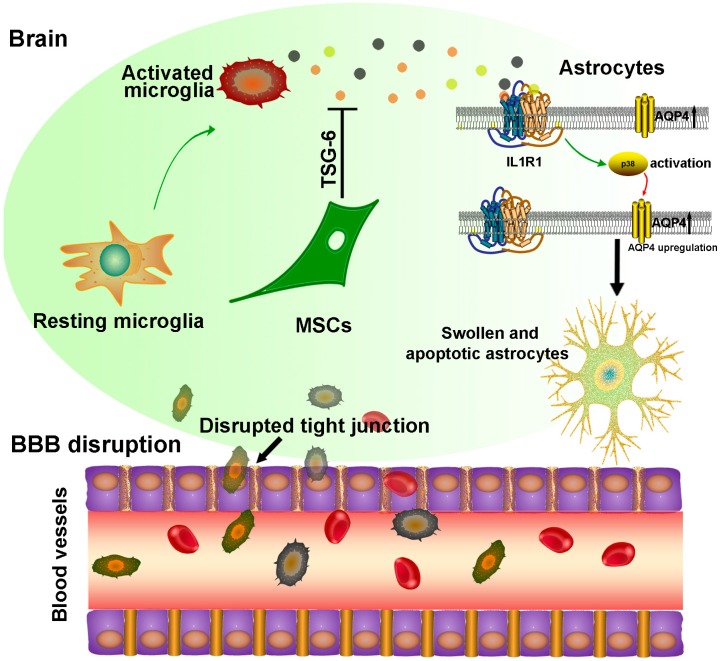
Mechanisms of MSCs in reducing ischemia-induced cerebral edema.

As a key player following brain injury, the inflammation response exacerbates the development of brain edema by disrupting BBB integrity [20,44]. Our study demonstrated that this process was highly associated with the upregulation of aquaporin-4 after cerebral ischemia. Using a transient ischemic model of the striatal transplantation of mesenchymal stem cells (MSCs), we illustrated that MSCs decreased inflammatory cytokines’ release from ischemia-activated microglia, thus restraining p38 phosphorylation and aquaporin-4 upregulation in astrocytes. Through this pathway, astrocyte apoptosis was reduced, and BBB was protected, leading to a better neurological outcome after ischemia. However, the receptor that mediates the activation of p38 in response to inflammatory cytokines was not under investigation. IL-1 receptor type 1 (IL1R1) may be involved in this process [61] (Figure 1). Even though we just examined the surviving state of MSCs after three days of transplantation, another study showed that the transplanted human MSCs expressed markers for astrocytes, oligodendroglia and neurons at six weeks after transplantation with improved functional performance in limb placement test [62]. Moreover, Bingchuan Xie with his colleagues demonstrated that human umbilical cord MSC transplantation in ischemic encephalopathy patients led to a better recovery without significant adverse effects [63], which represents a direct application in clinics. However, the clinical studies so far are still scarce, and more comprehensive evaluations are needed for further clinical application.

In cerebral ischemia, the resting microglia are activated, which leads to more inflammatory cytokine production, like IL1β (yellow dots), IL6 (black dots) and TNF-α (red dots). Mesenchymal stem cells (MSCs) are transplanted into the injured striatum within 20 min following reperfusion. Though secretion of TSG-6 or other unknown mechanisms [64], MSCs inhibit the release of inflammatory cytokines or blunt its activity. Since the interaction between the inflammatory cytokines and the IL1R1 receptor on the membrane of astrocytes leads to the phosphorylation of p38, upregulation of AQP4 and apoptosis of astrocytes, thereby, MSC treatment reduces the apoptotic astrocytes. As astrocyte-endothelial crosstalk is important to maintain the function of the BBB via inducing tight junction formation [65], through this pathway, BBB is protected, resulting in better neurological outcomes after ischemia.

MicroRNAs (miRNA) are single-stranded non-coding RNA molecules of ~22 nucleotides in length and function as gene expression regulators by binding to the 3′ untranslated region (UTR) of mRNA molecules. Recent studies have highlighted the role of miRNA as potential regulators in multiple pathophysiological processes [49,66,67]. Aquaporin-4 has been shown as a target gene of miRNA-29b in cerebral ischemia. Data from human blood samples revealed that miRNA-29b was significantly decreased in stroke patients, and poor outcomes were associated with miRNA-29b downregulation. In a mice study, we further demonstrated that the miR-29b level negatively correlated with aquaporin-4 expression, but had a positive correlation with the outcomes. Additionally, the dual-luciferase reporter system directly showed that aquaporin-4 was the target of miR-29b [49].

Together, with these studies, it could be optimistically stated that both stem cells and miRNAs can exert a potent regulating function on aquaporin-4 expression, which represents a novel tool for water balance intervention in brain insults.

## 6. Conclusions

Aquaporin-4 plays a crucial role in the edema process in different brain pathologies, and its interference seems to be important for the recovery in the acute phase after cerebral edema. However, the bimodal function of aquaporin-4 in the progression of cytotoxic and vasogenic edema has to be kept in mind when formulating strategies targeting aquaporin-4 in edema therapy. If taking the findings for transitioning from the cytotoxic type to the vasogenic type into consideration, pharmaceuticals, stem cells or miRNAs targeting aquaporin-4 are still full of challenges and have to be carefully considered in the effort toward any targeted interventions.

## Figures and Tables

**Table 1:** Biphasic function of AQP4 in cytotoxic and vasogenic edema.

Animal/Cell Type	Model	AQP4 Intervention Methods	AQP4 Reaction	Outcomes	Ref.
Mice	Intrastriatal ringer or quinolinic acid injection (cytotoxic edema)	-	AQP4 mRNA induction in hypertrophic astrocytes	BBB disruption	[47]
Cultured astrocytes	Fluid percussion injury (FPI) (cytotoxic edema)	AQP4 knockdown by siRNA	Dampened AQP4 induction	reduction in trauma-induced astrocyte swelling	[32]
Mice	Acute water intoxication and ischemic stroke (cytotoxic edema)	AQP4 global deletion	Complete deletion	Decreased hemispheric enlargement	[33]
Mice	Systemic hypoosmotic stress (cytotoxic edema)	AQP4 glial-conditional deletion	Conditional AQP4 knockout	Reduction in brain water uptake and a delayed postnatal resorption of brain water	[48]
Mice	Acute water intoxication (cytotoxic edema)	AQP4 glial-conditional overexpression	Overexpression	Increased brain swelling and intracranial pressure (ICP)	[37]
Mice	90-min transient middle cerebral artery occlusion (cytotoxic edema)	MSCs intracranial transplantation and AQP4 siRNA intracranial injection	DecreasedAQP4 induction by ischemia	Reduced brain edema and BBB leakage	[16]
Mice	Permanent middle cerebral artery occlusion (cytotoxic edema)	Lentivirus-miR-29b (LV-29b) intracranial injection	DecreasedAQP4 induction by ischemia	Reduced brain edema and BBB leakage	[49]
Mice	I.P. injection of distilled water and 8-deamino-arginine vasopressin (cytotoxic edema)	Dystrophin global deletion	AQP4 mislocalization	Delayed onset of brain edema	[50]
Mice	Artificial cerebrospinal fluid (vasogenic edema)	AQP4 global deletion	Complete deletion	Increased brain swelling and ICP	[39]
Mice	Subarachnoid hemorrhage (vasogenic edema)	AQP4 global deletion	Complete deletion	increased brain water content and intracranial pressure	[51]

## Abbreviations

AQP4: aquaporin-4
AZA: acetazolamide
BBB: blood-brain barrier
CBF: cerebral blood flow
CSF: cerebrospinal fluid
MSCs: mesenchymal stem cells
ICP: intracranial pressure

## References

[1] Basarslan S.K., Göçmez C., Kamaşak K., Ekici M.A., Ulutabanca H., Doğu Y., Menkü A. (2015). The effects of erythropoietin, dextran and saline on brain edema and lipid peroxidation in experimental head trauma. Ulus Travma Acil Cerrahi Derg..

[2] Hossmann K.A. (1998). Experimental models for the investigation of brain ischemia. Cardiovasc. Res..

[3] Papadopoulos M.C., Saadoun S., Binder D.K., Manley G.T., Krishna S., Verkman A.S. (2004). Molecular mechanisms of brain tumor edema. Neuroscience.

[4] Rasmussen T., Gulati D.R. (1962). Cortisone in the treatment of postoperative cerebral edema. J. Neurosurg..

[5] Zador Z., Stiver S., Wang V., Manley G.T. (2009). Role of aquaporin-4 in cerebral edema and stroke. Handb. Exp. Pharmacol..

[6] Denker B.M., Smith B.L., Kuhajda F.P., Agre P. (1988). Identification, purification, and partial characterization of a novel Mr 28,000 integral membrane protein from erythrocytes and renal tubules. J. Biol. Chem..

[7] Takata K., Matsuzaki T., Tajika Y. (2004). Aquaporins: Water channel proteins of the cell membrane. Prog. Histochem. Cytochem..

[8] Bond M.R. (1978). Assessment of outcome following severe closed head injury. Scott. Med. J..

[9] Go K.G. (1984). Pathophysiological aspects of brain edema. Clin. Neurol. Neurosurg..

[10] Klatzo I. (1967). Neuropathological aspects of brain edema. J. Neuropathol. Exp. Neurol..

[11] Milhorat T.H. (1992). Classification of the cerebral edemas with reference to hydrocephalus and pseudotumor cerebri. Childs Nerv. Syst..

[12] Liu Y., Tang G.H., Sun Y.H., Lin X.J., Wei C., Yang G.-Y., Liu J.R. (2013). The protective role of Tongxinluo on blood-brain barrier after ischemia-reperfusion brain injury. J. Ethnopharmacol..

[13] Taniguchi M., Yamashita T., Kumura E., Tamatani M., Kobayashi A., Yokawa T., Maruno M., Kato A., Ohnishi T., Kohmura E. (2000). Induction of aquaporin-4 water channel mRNA after focal cerebral ischemia in rat. Brain Res. Mol. Brain Res..

[14] Cross J.L., Meloni B.P., Bakker A.J., Lee S., Knuckey N.W. (2010). Modes of Neuronal Calcium Entry and Homeostasis following Cerebral Ischemia. Stroke Res. Treat..

[15] Fu X., Li Q., Feng Z., Mu D. (2007). The roles of aquaporin-4 in brain edema following neonatal hypoxia ischemia and reoxygenation in a cultured rat astrocyte model. Glia.

[16] Tang G., Liu Y., Zhang Z., Lu Y., Wang Y., Huang J., Li Y., Chen X., Gu X., Wang Y. (2014). Mesenchymal stem cells maintain blood-brain barrier integrity by inhibiting aquaporin-4 upregulation after cerebral ischemia. Stem Cells.

[17] Stokum J.A., Kurland D.B., Gerzanich V., Marc Simard J. (2015). Mechanisms of Astrocyte-Mediated Cerebral Edema. Neurochem. Res..

[18] Wolburg H., Noell S., Fallier-Becker P., Mack A.F., Wolburg-Buchholz K. (2012). The disturbed blood-brain barrier in human glioblastoma. Mol. Asp. Med..

[19] Steiner E., Enzmann G.U., Lin S., Ghavampour S., Hannocks M.J., Zuber B., Rüegg M.A., Sorokin L., Engelhardt B. (2012). Loss of astrocyte polarization upon transient focal brain ischemia as a possible mechanism to counteract early edema formation. Glia.

[20] Huang J., Li Y., Tang Y., Tang G., Yang G.Y., Wang Y. (2013). CXCR4 antagonist AMD3100 protects blood-brain barrier integrity and reduces inflammatory response after focal ischemia in mice. Stroke.

[21] Rash J.E., Yasumura T. (1999). Direct immunogold labeling of connexins and aquaporin-4 in freeze-fracture replicas of liver, brain, and spinal cord: Factors limiting quantitative analysis. Cell Tissue Res..

[22] Papadopoulos M.C., Verkman A.S. (2007). Aquaporin-4 and brain edema. Pediatr. Nephrol..

[23] Iacovetta C., Rudloff E., Kirby R. (2012). The role of aquaporin 4 in the brain. Vet. Clin. Pathol..

[24] Jackson I.J., Snodgrass S.R. (1955). Peritoneal Shunts in the Treatment of Hydrocephalus and Increased Intracranial Pressure—A 4-Year Survey of 62 Patients. J. Neurosurg..

[25] Gomes D., Agasse A., Thiébaud P., Delrotc S., Gerós H., Chaumont F. (2009). Aquaporins are multifunctional water and solute transporters highly divergent in living organisms. Biochim. Biophys. Acta.

[26] Yu J., Yool A.J., Schulten K., Tajkhorshid E. (2006). Mechanism of gating and ion conductivity of a possible tetrameric pore in aquaporin-1. Structure.

[27] Hiroaki Y., Tania K., Kamegawa A., Gyobu N., Nishikawa K., Suzuki H., Walz T., Sasaki S., Mitsuoka K., Kimura K. (2006). Implications of the aquaporin-4 structure on array formation and cell adhesion. J. Mol. Biol..

[28] Musa-Aziz R., Chen L.-M., Pelletier M.F., Boron W.F. (2009). Relative CO_2_/NH_3_ selectivities of AQP1, AQP4, AQP5, AmtB, and RhAG. Proc. Natl. Acad. Sci. USA.

[29] Rash J.E., Yasumura T., Sue Hudson C., Agre P., Nielsen S. (1998). Direct immunogold labeling of aquaporin-4 in square arrays of astrocyte and ependymocyte plasma membranes in rat brain and spinal cord. Proc. Natl. Acad. Sci. USA.

[30] Ma T., Yang B., Gillespie A., Carlson E.J., Epstein C.J., Verkman A.S. (1997). Generation and phenotype of a transgenic knockout mouse lacking the mercurial-insensitive water channel aquaporin-4. J. Clin. Investig..

[31] Saadoun S., Papadopoulos M.C., Watanabe H., Yan D., Manley G.T., Verkman A.S. (2005). Involvement of aquaporin-4 in astroglial cell migration and glial scar formation. J. Cell Sci..

[32] Rao K.V., Reddy P.V., Curtis K.M., Norenberg M.D. (2011). Aquaporin-4 expression in cultured astrocytes after fluid percussion injury. J. Neurotrauma.

[33] Manley G.T., Fujimura M., Ma T., Noshita N., Filiz F., Bollen A.W., Chan P., Verkman A.S. (2000). Aquaporin-4 deletion in mice reduces brain edema after acute water intoxication and ischemic stroke. Nat. Med..

[34] Zeng X.N., Xie L.L., Liang R., Sun X.L., Fan Y., Hu G. (2012). AQP4 knockout aggravates ischemia/reperfusion injury in mice. CNS Neurosci. Ther..

[35] Sun M.C., Honey C.R., Berk C., Wong N.L., Tsui J.K. (2003). Regulation of aquaporin-4 in a traumatic brain injury model in rats. J. Neurosurg..

[36] Papadopoulos M.C., Verkman A.S. (2005). Aquaporin-4 gene disruption in mice reduces brain swelling and mortality in pneumococcal meningitis. J. Biol. Chem..

[37] Yang B., Zador Z., Verkman A.S. (2008). Glial cell aquaporin-4 overexpression in transgenic mice accelerates cytotoxic brain swelling. J. Biol. Chem..

[38] Marmarou A., Hochwald G., Nakamura T., Tanaka K., Weaver J., Dunbar J. (1994). Brain edema resolution by CSF pathways and brain vasculature in cats. Am. J. Physiol..

[39] Papadopoulos M.C., Manley G.T., Krishna S., Verkman A.S. (2004). Aquaporin-4 facilitates reabsorption of excess fluid in vasogenic brain edema. FASEB J..

[40] Thrane A.S., Rappold P.M., Fujita T., Torres A., Bekar L.K., Takano T., Peng W., Wang F., Rangroo Thrane V., Enger R. (2011). Critical role of aquaporin-4 (AQP4) in astrocytic Ca^2+^ signaling events elicited by cerebral edema. Proc. Natl. Acad. Sci. USA.

[41] Svitkina T.M., Borisy G.G. (1999). Progress in protrusion: The tell-tale scar. Trends Biochem. Sci..

[42] Small J.V., Stradal T., Vignal E., Rottner K. (2002). The lamellipodium: Where motility begins. Trends Cell Biol..

[43] Tomas-Camardiel M., Venero J.L., De Pablos R.M., Rite I., Machado A., Cano J. (2004). In vivo expression of aquaporin-4 by reactive microglia. J. Neurochem..

[44] Han X., Fink M.P., Delude R.L. (2003). Proinflammatory cytokines cause NO*-dependent and -independent changes in expression and localization of tight junction proteins in intestinal epithelial cells. Shock.

[45] Tourdias T., Mori N., Dragonu I., Cassagno N., Boiziau C., Aussudre J., Brochet B., Moonen C., Petry K.G., Dousset V. (2011). Differential aquaporin 4 expression during edema build-up and resolution phases of brain inflammation. J. Neuroinflamm..

[46] Nielsen S., Nagelhus E.A., Amiry-Moghaddam M., Bourque C., Agre P., Ottersen O.P. (1997). Specialized membrane domains for water transport in glial cells: High-resolution immunogold cytochemistry of aquaporin-4 in rat brain. J. Neurosci..

[47] Vizuete M.L., Venero J.L., Vargas C., Ilundain A.A., Echevarra M., Machado A., Cano J. (1999). Differential upregulation of aquaporin-4 mRNA expression in reactive astrocytes after brain injury: Potential role in brain edema. Neurobiol. Dis..

[48] Haj-Yasein N.N., Vindedal G.F., Eilert-Olsen M., Gundersen G.A., Skare Ø., Laake P., Klungland A., Thorén A.E., Burkhardt J.M., Ottersen O.P. (2011). Glial-conditional deletion of aquaporin-4 (Aqp4) reduces blood-brain water uptake and confers barrier function on perivascular astrocyte endfeet. Proc. Natl. Acad. Sci. USA.

[49] Wang Y., Huang J., Ma Y., Tang G., Liu Y., Chen X., Zhang Z., Zeng L., Wang Y., Ouyang Y.B. (2015). MicroRNA-29b is a therapeutic target in cerebral ischemia associated with aquaporin 4. J. Cereb. Blood Flow Metab..

[50] Vajda Z., Pedersen M., Füchtbauer E.-M., Wertz K., Stødkilde-Jørgensen H., Sulyok E., Dóczi T., Neely J.D., Agre P., Frøkiær J. (2002). Delayed onset of brain edema and mislocalization of aquaporin-4 in dystrophin-null transgenic mice. Proc. Natl. Acad. Sci. USA.

[51] Tait M.J., Saadoun S., Bell B.A., Verkman A.S., Papadopoulos M.C. (2010). Increased brain edema in *aqp4*-null mice in an experimental model of subarachnoid hemorrhage. Neuroscience.

[52] Huber V.J., Tsujita M., Yamazaki M., Sakimura K., Nakada T. (2007). Identification of arylsulfonamides as Aquaporin 4 inhibitors. Bioorg. Med. Chem. Lett..

[53] Huber V.J., Tsujita M., Kwee I.L., Nakada T. (2009). Inhibition of aquaporin 4 by antiepileptic drugs. Bioorg. Med. Chem..

[54] Igarashi H., Huber V.J., Tsujita M., Nakada T. (2011). Pretreatment with a novel aquaporin 4 inhibitor, TGN-020, significantly reduces ischemic cerebral edema. Neurol. Sci..

[55] Brines M., Cerami A. (2005). Emerging biological roles for erythropoietin in the nervous system. Nat. Rev. Neurosci..

[56] Digicaylioglu M., Lipton S.A. (2001). Erythropoietin-mediated neuroprotection involves cross-talk between Jak2 and NF-kappaB signalling cascades. Nature.

[57] Juul S. (2012). Neuroprotective role of erythropoietin in neonates. J. Matern. Fetal Neonatal Med..

[58] Nichol A., French C., Little L., Haddad S., Presneill J., Arabi Y., Bailey M., Cooper D.J., Duranteau J., Huet O. (2015). Erythropoietin in traumatic brain injury (EPO-TBI): A double-blind randomised controlled trial. Lancet.

[59] Gunnarson E., Song Y., Kowalewski J.M., Brismar H., Brines M., Cerami A., Andersson U., Zelenina M., Aperia A. (2009). Erythropoietin modulation of astrocyte water permeability as a component of neuroprotection. Proc. Natl. Acad. Sci. USA.

[60] Cruz-Orengo L., Holman D.W., Dorsey D., Zhou L., Zhang P., Wright M., McCandless E.E., Patel J.R., Luker G.D., Littman D.R. (2011). CXCR7 influences leukocyte entry into the CNS parenchyma by controlling abluminal CXCL12 abundance during autoimmunity. J. Exp. Med..

[61] Ito H., Yamamoto N., Arima H., Hirate H., Morishima T., Umenishi F., Tada T., Asai K., Katsuya H., Sobue K. (2006). Interleukin-1beta induces the expression of aquaporin-4 through a nuclear factor-kappaB pathway in rat astrocytes. J. Neurochem..

[62] Zhao L.R., Duan W.M., Reyes M., Keene C.D., Verfaillie C.M., Low W.C. (2002). Human bone marrow stem cells exhibit neural phenotypes and ameliorate neurological deficits after grafting into the ischemic brain of rats. Exp. Neurol..

[63] Xie B., Gu P., Wang W., Dong C., Zhang L., Zhang J., Liu H., Qiu F., Han R., Zhang Z. (2016). Therapeutic effects of human umbilical cord mesenchymal stem cells transplantation on hypoxic ischemic encephalopathy. Am. J. Transl. Res..

[64] Redell J.B., Moore A.N., Ward N.H., Hergenroeder G.W., Dash P.K. (2010). Human traumatic brain injury alters plasma microRNA levels. J. Neurotrauma.

[65] Redell J.B., Liu Y., Dash P.K. (2009). Traumatic brain injury alters expression of hippocampal microRNAs: Potential regulators of multiple pathophysiological processes. J. Neurosci. Res..

[66] Prockop D.J., Oh J.Y. (2012). Mesenchymal stem/stromal cells (MSCs): Role as guardians of inflammation. Mol. Ther..

[67] Arthur F.E., Shivers R.R., Bowman P.D. (1987). Astrocyte-mediated induction of tight junctions in brain capillary endothelium: An efficient in vitro model. Brain Res..

